# Enhancement of phycocyanin productivity and thermostability from *Arthrospira platensis* using organic acids

**DOI:** 10.1186/s12934-023-02256-2

**Published:** 2023-12-05

**Authors:** Mohamed Gomaa, Shimaa Abdelmohsen Ali, Awatief F. Hifney

**Affiliations:** Botany & Microbiology Department, Faculty of Science, Assiut University, Assiut, 71516 Egypt

**Keywords:** Phycobiliproteins, Citric acid, Thermodynamics, Fed-batch cultivation, Food-grade phycocyanin

## Abstract

Intracellular hyperaccumulation of phycocyanin (PC) and its high susceptibility to degradation at higher temperatures are major challenging problems associated with its production from cyanobacteria. The present study evaluated different concentrations of organic acids (1, 2, and 3 mM) (citric acid, acetic acid, succinic acid, fumaric acid, and oxalic acid) under fed-batch mode on the biomass and phycobiliproteins’ production from *Arthrospira platensis*. Besides they were evaluated at 2.5–7.5 mM as preservative to stabilize PC at high temperatures. The incorporation of 3 mM of succinic acid into the cultivation medium enhanced the biomass and PC productivity to 164.05 and 26.70 mg L^−1 ^day^−1^, which was ~ 2- and threefold higher than control, respectively. The produced PC in this treatment was food-grade with a 2.2 purity ratio. The use of organic acids also enhanced the thermal stability of PC. Citric acid (7.5 mM) markedly promoted the half-life values of PC to 189.44 min compared to 71.84 min in the control. The thermodynamic analysis confirmed higher thermostability of PC in the presence of organic acids and indicated the endothermic and non-spontaneity of the thermal denaturation process. The findings of the present study confirmed that organic acids could be utilized as cost effective and sustainable compounds for promoting not only phycobiliproteins’ production but also the thermostability of PC for potential application in food industry.

## Introduction

*Arthrospira* (*Spirulina*) is an economically important genus of Cyanobacteria, which is generally regarded as safe (GRAS) without any side-effects on human health. It has promising positive effects against various diseases such cancer, diabetes, anemia, obesity, as well as antibacterial, antiviral, and anti-inflammatory activities [1]. Accordingly, it is widely exploited to produce nutrient-rich food and fodder, pharmaceutical and cosmetical products, food additives and colorants [2]. (*Arthrospira platensis*) *A. platensis* is a common species of Cyanobacteria, which consists of multi-cellular spirally twisted filaments [3]. The main proteinaceous constituents in *A. platensis* with high economic importance are phycobiliproteins (phycocyanin, allophycocyanin, and phycoerythrin). The cyanobacterial cell is usually rich in phycocyanin (PC) in relation to other phycobiliproteins. PC has bright blue color, thus is mainly utilized as a colorant for several food and cosmetical products. Besides, it is generally regarded as a potent therapeutic agent with versatile bioactivities such as antioxidant, anticancer, and anti-inflammatory properties [4, 5]. The market price of PC depends on its purity and the price of food-grade product is around $130 *g*^−1^ [6]. Furthermore, the global annual market of PC was estimated at $155.3 million in 2020, which is expected to increase to $409.8 million by 2030 [6].

Higher productivity of *A. platensis* biomass and hyperaccumulation of PC are generally important to achieve cost-effectiveness and economic feasibility. The microalgal growth and biosynthesis of metabolites can be altered under different abiotic factors such as light, nutrient enrichment or deficiency, temperature, etc. [7]. Different organic acids such as citric acid, succinic acid, fumaric acid, and oxalic acid are well known intermediates of the tricarboxylic acid (TCA) cycle [8]. Accordingly, the supplementation of these compounds into the culture medium at small appropriate quantities can boost the microalgal biomass production as well as the biosynthesis of certain metabolites such as PC. However, little information is available in the literature regarding the effects of these compounds on cyanobacterial growth. The incorporation of succinic acid at 7.5 mM into the cultivation medium as a metabolic stressor have been reported to promote biomass and PC production of *A. platensis* [9]*.* In another study, an enhancement of the biomass and astaxanthin production of the green microalga *Haematococcus pluvialis* was observed under fed-batch cultivation using succinic acid [10]. To the best of our knowledge, no attempts have been performed to compare the effects of different organic acids intermediates on cyanobacterial growth and PC production under fed-batch cultivation. Generally, these organic acids are readily available as inexpensive synthetic compounds, making their use in the cultivation of microalgae and cyanobacteria economically feasible.

On the other hand, the main disadvantage of PC, which limits its potential industrial applications, is its high sensitivity and denaturation by heat [11, 12]. Thus, various attempts have been carried out to increase the thermostability of PC through preservatives such CaCl_2_, sugars and citric acid. According to our knowledge, there is insufficient comparative research on the use of different organic acids as preservatives for PC at high temperatures [11, 13, 14]. It is also crucial to investigate different kinetic and thermodynamic parameters during the thermal color loss of PC for effective application in various industries.

The aim of the present study was set to investigate the simultaneous enhancement of biomass and PC production of *A. platensis* under fed-batch cultivation using organic acids namely oxalic acid, acetic acid, citric acid, fumaric acid and succinic acid. Furthermore, these organic acids were tested as possible preservatives for enhancing the thermostability of PC. The best organic acids to promote the productivity and thermostability of PC were determined.

## Material and methods

### Cyanobacterial growth conditions

*Arthrospira* (*Spirulina*) *plantensis* was isolated from the farm of faculty of agriculture, Assiut university. *A. platensis* was cultivated in Zarrouk’s medium [15] consisting of (*g* L^−1^): NaHCO_3_, 16.8; NaNO_3_, 2.5; CaCl_2_.2H_2_O, 0.04; MgSO_4_.7H_2_O, 0.2; K_2_HPO_4_, 0.5; K_2_SO_4_, 1; NaCl, 1; Na_2_EDTA, 0.08; FeSO_4_.7H_2_O, 0.01; and micronutrients (mg L^−1^): H_3_BO_3_, 2.86; MnCl_2_.4H_2_O, 1.81; ZnSO_4_, 0.222; Na_2_Mo_7_O_4_, 0.0177; CuSO_4_.5H_2_O, 0.079 (pH 10.5). The cyanobacterium was grown in a 2000 mL flask under continuous illumination using fluorescent lamps at 48.4 µmolm^−2^s^−1^ and gassed with sterile air provided by air pump at 25 ℃.

### Cyanobacterial cultivation with different organic acids

Five different organic acids namely oxalic acid, acetic acid, citric acid, fumaric acid and succinic acid were evaluated separately as an additional carbon source to enhance the growth of *A. platensis* and its phycobiliproteins’ accumulation. In order to avoid the toxic effects of these acids on the cyanobacterial growth, the treatment was performed by fed-batch cultivation. Accordingly, the substrates were fed into the cyanobacterial culture at 3-day intervals to reach a final concentration of 1, 2, or 3 mM in the culture medium. At the beginning of the experiment, a 7-day old cyanobacterial culture was harvested by centrifugation and used to inoculate 200 mL autoclaved Zarrouk’s medium containing different concentrations (1, 2, or 3 mM) of each organic acid. The experiment was performed in 250 mL Erlenmeyer flasks containing 200 mL culture. The initial cyanobacterial concentration was set to 0.2 optical density at 750 nm, which corresponds to 0.78 *g* L^−1^ dry cell weight (DCW). This initial cell concentration was selected based on a preliminary experiment to enhance both cell growth and phycocyanin content. Cell growth in Zarrouk’s medium without the addition of organic acids served as a control. The cyanobacterial growth was proceeded under continuous illumination using fluorescent lamps at 48.4 µmolm^−2^s^−1^ and gassed with sterile air provided by air pump at 25 ℃. The experiments were terminated after 12 days since phycocyanin productivity decreased with longer cultivation periods.

### Determination of cyanobacterial growth and biomass productivity

At predetermined time interval, the microalgal growth was monitored by measuring optical density at 750 nm using a spectrophotometer (Unico UV-2100, USA). A relationship between optical density and dry cell weights (DCW, *g* L^−1^) was established using regression analysis. The DCW of the microalgal cells was determined after collecting the cells by centrifugation (3800 *g*, 10 min) followed by oven drying (60 ℃). Biomass productivity (BP, mg L^−1^ day^−1^) was calculated using the following equation:

1 $$Biomass \, productivity= \frac{{X}_{1}- {X}_{0}}{{t}_{1}-{t}_{0}}$$

where *X*_*1*_ and *X*_*0*_ represent the final and initial biomass concentration at the end of the experiment (*t*_*1*_ = 12 day) and the beginning (*t*_*0*_ = 0), respectively.

### Phycocyanin extraction and concentration analysis

At the end of the growth period, the cyanobacterial cells were harvested by centrifugation (3800 *g*, 10 min). phycobiliproteins were extracted by repeated freezing and thawing method [16]. The cyanobacterial biomass was frozen at − 19 ℃ for 1 day then thawed at room temperature for one hour, this process was repeated four times. Then the extracted pigments were dissolved in 15 mL of phosphate buffer (0.1 M, pH 7). The residual cell debris was then removed by centrifugation. The concentration of phycocyanin (PC), phycoerythrin (PE) and allophycocyanin (APC) in the supernatant was determined spectrophotometrically by measuring the absorbance of the crude extract at 280, 562, 620, 652 nm respectively. The concentrations of these pigments was estimated using the following equations [17]:

2 $${\text{PC }}(mg\,mL^{- 1} ) = (A_{(620)} - 0.474 \, \times A_{6} 52)/5.34$$

3 $${\text{APC}}\, \left( {mg\,mL^{ - 1} } \right) = \frac{{A_{652} - 0.208{ } \times { }A_{620} }}{5.09}$$

4 $${\text{PE}} \,\left( {mg\,mL^{ - 1} } \right) = \frac{{A_{562} - 2.41{ } \times { }PC - 0.849{ } \times { }APC}}{9.62}$$

where A refers to the absorbance at the corresponding wavelength.

The phycocyanin purity ratio (purity index) was calculated by the following ratio [18]:

5 $${\text{Purity PC}} = \frac{{A_{620} }}{{A_{280} }}$$

### Effect of organic acids on the thermostability of phycocyanin

In this experiment, the organic acids viz*.* oxalic acid, acetic acid, citric acid, fumaric acid and succinic acid were evaluated as preservatives to enhance the thermostability of crude phycocyanin extract from *A. plantensis.* The thermo-kinetic stability of phycobiliprotein extract was evaluated by preparing a known concentration of crude PC in phosphate buffer (0.1 M, pH 7.0). Then, the samples were treated with different organic acids at different final concentrations (2.5, 5, and 7.5 mM), followed by incubation at different temperatures (35, 45, 55, 65, and 75 ℃) for 30-, 60-, and 90-min. The stability of PC was monitored for each treatment by measuring the absorbance using a spectrophotometer as described in SubSect. "Phycocyanin extraction and concentration analysis".

### Kinetics and quantification of PC degradation

The time-dependent degradation of PC at different temperatures in the presence or absence of organic acids was quantified using a first-order exponential equation to estimate the degradation rate constant (*K*, min^−1^) using the following equation [19]:

6 $${\text{Ct}} = {\text{C}}_{0} \,e^{ - Kt}$$

7 $$ln\,\left( {\frac{{C_{t} }}{{C_{0} }}} \right) = - kt$$

where *C*_*t*_ is the concentration of PC after time t, *C*_*0*_ is its initial concentration. The *K* values were obtained by linear regression analysis of the plot ln *(*c_t_*/*c_0_*)* vs.* t.*

Additionally, the PC thermostability was analyzed by using the following half-life equation to calculate the time required for the degradation of 50% of the initial PC concentration (*t*_*1/2*_) [20]:

8 $${t}_{1/2}=\frac{0.693}{K}$$

### Thermodynamics of PC degradation

The following Arrhenius equation was used to calculate the activation energy of the degradation reaction (*E*, kJ mol^−1^) [21],

9 $$K={K}_{0}{e}^{-E/RT}$$

where *K* is the degradation rate constant, *K*_*0*_ is the frequency factor, *R* is the universal gas constant (8.3145 J^−1^ mol^−1^ K^−1^), and *T* is the absolute temperature (K). The slope of Arrhenius plot (ln K vs. 1/T) provides an estimation of *E* values, where slope =  − *E*/*R. *The values of the *K* and *E* were used to calculate the variation of enthalpy of inactivation (△*H*°, KJ mol^−1^), Gibb’s free energy (△*G*°, KJ mol^−1^), and variation of entropy (△*S*°, KJ mol^−1^) using the following equations [21]:

10 $$\Delta H^\circ =E-RT$$

11 $$\Delta G^\circ =-RT\mathrm{ln}\left(\frac{Kh}{{K}_{B}T}\right)$$

12 $$\Delta S^\circ =\frac{\Delta H^\circ -\Delta G^\circ }{\mathrm{T}}$$

where h is the Planck constant (11.04 × 10^−34^ J min), and KB is the Boltzmann constant (1.3806 × 10^−23^ J K^−1^).

### Cyanobacterial growth modeling and Statistical analysis

The kinetics of *A. platensis* growth were modeled using the modified logistic model using the following equation [22, 23],

13 $$X\left(t\right)={X}_{0}+ \frac{({X}_{max}-{X}_{0})}{1+exp \left\{\left(\frac{4{\mu }_{max}}{{X}_{max}-{X}_{0}}\right)\left(\lambda -t\right)+2\right\}}$$

where *X(t)*, *X*_*0*_, *X*_*max*_ are the time dependent increase in *A. platensis* biomass (*g *L^−1^), the initial biomass concentration and the maximum biomass concentration, respectively. While *µ*_*max*_ refers to the maximum growth rate (day^−1^) and *λ* indicates the lag time (day). The equation was solved by applying solver function in Excel 2016 software by minimizing the following error equation of root mean square error (RMSE):

14 $$RMSE= \sqrt{\frac{\sum_{t=1}^{N}{({X\left(t\right)}_{calc}- {X\left(t\right)}_{exp})}^{2}}{N}}$$

where *X(t)*_*calc*_ and *X(t)*_*exp*_ represents the calculated and the experimental *A. platensis* biomass at time *t* and *N* is the number of experimental points.

The significant differences between treatments at *p* < 0.05 were estimated using analysis of variance (ANOVA) followed by post-hoc Fisher’s least square difference (LSD) test using GNU PSPP statistical software (V 1.6.2).

## Results

### Effect of different organic acids on *A. platensis* growth

**Fig. 1:**
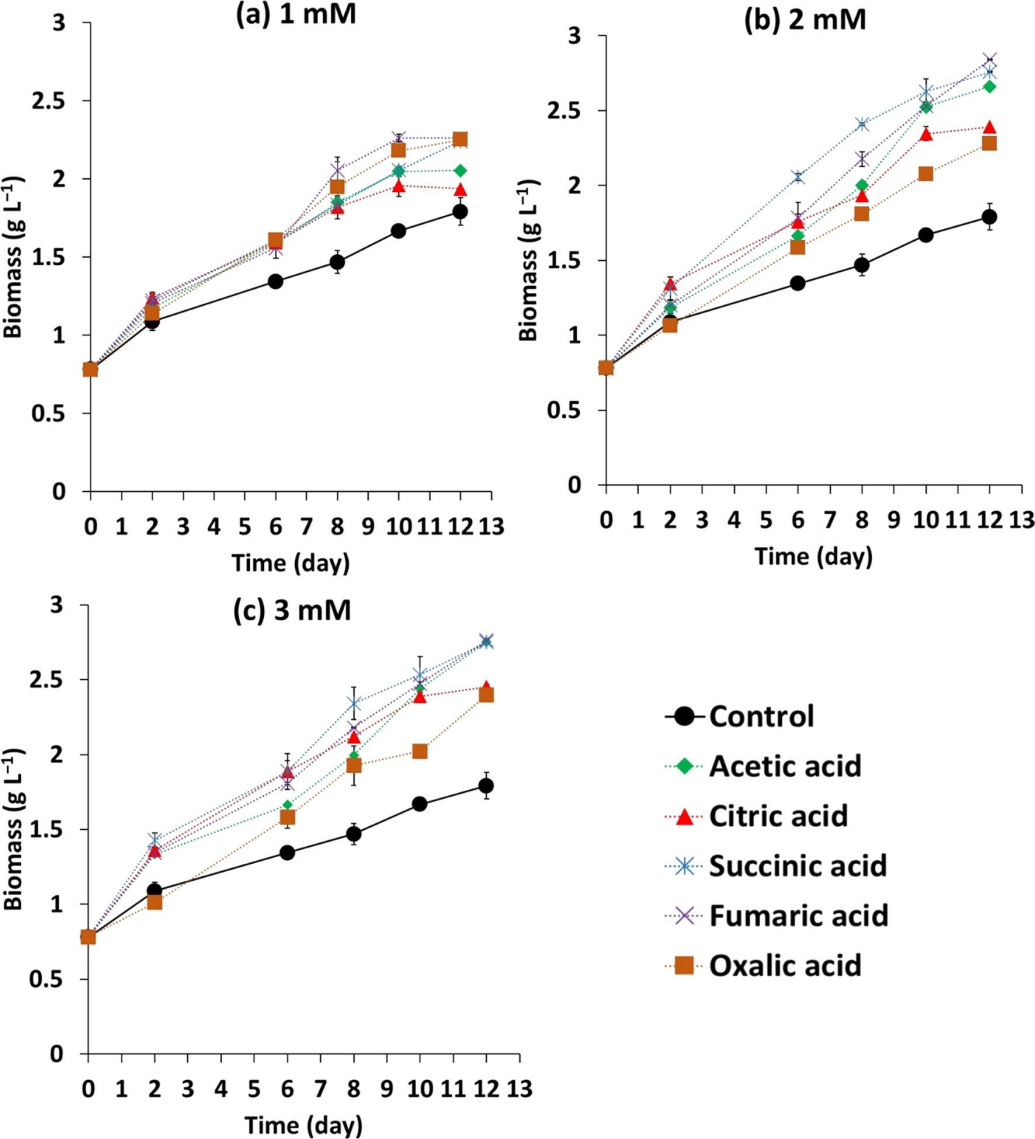
Growth curves of *A. platensis* under fed-batch supplementation of different concentrations of organic acids (**a**) 1 mM, (**b**) 2 mM, and (**c**) 3 mM at 3-day intervals. The incorporation of different organic acids was at day 0, 3, 6, and 9

*A. platensis* was cultivated in Zarrouk’s medium supplemented with different concentrations of different organic acids using fed-batch cultivation. The time-dependent growth of *A. platensis* under different treatments was depicted in Fig. 1. The tested organic acids and their different concentrations exhibited a remarkable promotion of *A. platensis* growth compared to the control. Furthermore, increasing organic acid concentration from 1 mM to 2 and/or 3 mM during the fed-batch cultivation enhanced the cyanobacterial biomass productivity (Table 1). Feeding the cyanobacterial culture with 2 mM of fumaric acid every 3-days exhibited higher biomass productivity of 171.56 ± 0.20 mg L^−1^ day^−1^ than other treatments. This value was twofold higher than the control without organic acids (84.15 ± 6.0 mg L^−1^ day^−1^) (Table 1). Additionally, the supplementation of 3 mM of acetic acid, succinic acid, or fumaric acid or 2 mM succinic acid showed also a marked increase in the cyanobacterial biomass productivity, and the enhancement was around 1.95-fold higher than the organic acid-less control.

**Table 1:** Effect of different organic acids on the biomass productivity (BP, mg L^−1^ day^−1^) and the contents (% w/w) and productivities (mg L^−1^ day^−1^) of phycocyanin (PC), phycoerythrin (PE), and allophycocyanin (APC) as well as PC purity ratio (A_620_/ A_280_)

Treatment	Biomass productivity	PC			PE		APC	
		Content	Productivity	Purity ratio	Content	Productivity	Content	Productivity
Control	84.15 ± 6.00^**a**^	16.92 ± 0.44^**a**^	1.42 ± 0.152^**a**^	0.88 ± 0.004^**b**^	3.42 ± 0.27^**b**^	0.29 ± 0.003^**a**^	4.66 ± 0.26^**d**^	0.39 ± 0.005^**b**^
AA (1 mM)	106.25 ± 0.80^**c**^	24.55 ± 0.37^** g**^	2.61 ± 0.059^**e**^	0.84 ± 0.001^**ab**^	4.58 ± 0.02^** fg**^	0.49 ± 0.006^**c**^	4.05 ± 0.02^**bc**^	0.43 ± 0.005^**c**^
AA (2 mM)	156.54 ± 1.00^**f**^	24.02 ± 0.01^**f**^	3.76 ± 0.023^** h**^	1.74 ± 0.004^**f**^	4.10 ± 0.06^**d**^	0.64 ± 0.014^**i**^	9.37 ± 0.05^**i**^	1.54 ± 0.018^**j**^
AA (3 mM)	164.19 ± 0.20^** g**^	22.40 ± 0.32^**d**^	3.68 ± 0.048^** h**^	1.92 ± 0.004^**gh**^	4.44 ± 0.04^**ef**^	0.73 ± 0.007^** k**^	3.38 ± 0.01^**ij**^	0.33 ± 0.004^** l**^
CA (1 mM)	96.33 ± 0.001^**b**^	19.30 ± 0.01^**c**^	1.86 ± 0.001^**b**^	1.36 ± 0.002^**a**^	4.12 ± 0.01^**d**^	0.40 ± 0.001^**b**^	7.38 ± 0.03^**a**^	0.99 ± 0.003^**a**^
CA (2 mM)	134.16 ± 0.60^**e**^	24.11 ± 0.03^** fg**^	3.23 ± 0.018^**f**^	2.01 ± 0.001^** h**^	6.24 ± 0.04^**j**^	0.84 ± 0.009^**n**^	7.38 ± 0.04^** g**^	0.99 ± 0.010^** g**^
CA (3 mM)	139.26 ± 1.40^**e**^	22.98 ± 0.01^**e**^	3.20 ± 0.032^**f**^	1.59 ± 0.001^**e**^	4.53 ± 0.01 fg	0.63 ± 0.005^**i**^	7.09 ± 0.06^**f**^	0.99 ± 0.019^** g**^
SA (1 mM)	121.83 ± 2.00^**d**^	18.96 ± 0.07^**c**^	2.31 ± 0.047^**d**^	1.03 ± 0.002^**c**^	4.57 ± 0.01 fg	0.56 ± 0.009^** fg**^	3.44 ± 0.01^**a**^	0.42 ± 0.006^**c**^
SA (2 mM)	164.62 ± 0.40^** g**^	25.45 ± 0.27^** h**^	4.19 ± 0.035^**j**^	1.86 ± 0.161^** fg**^	4.27 ± 0.07^**de**^	0.70 ± 0.009^**j**^	9.58 ± 0.02^**j**^	1.58 ± 0.001^** m**^
SA (3 mM)	164.05 ± 0.40^** g**^	26.70 ± 0.01^**i**^	4.38 ± 0.010^** k**^	2.20 ± 0.091^**i**^	4.74 ± 0.01^**gi**^	0.78 ± 0.001^** l**^	10.34 ± 0.00^** k**^	1.70 ± 0.004^**n**^
FA (1 mM)	123.53 ± 0.40^**d**^	18.35 ± 0.04^**b**^	2.27 ± 0.003^**d**^	1.03 ± 0.001^**c**^	4.57 ± 0.01^** fg**^	0.56 ± 0.003^**gh**^	4.23 ± 0.01^**c**^	0.52 ± 0.003^**e**^
FA (2 mM)	171.56 ± 0.20^** h**^	23.29 ± 0.12^**e**^	3.99 ± 0.016^**i**^	2.26 ± 0.101^**i**^	3.13 ± 0.02^**a**^	0.54 ± 0.003^**e**^	8.77 ± 0.05^** h**^	1.51 ± 0.016^** k**^
FA (3 mM)	165.04 ± 1.00^** g**^	24.55 ± 0.07^** g**^	4.05 ± 0.014^**i**^	1.32 ± 0.054^**d**^	4.84 ± 0.01^**i**^	0.80 ± 0.006^** m**^	7.43 ± 0.05^** g**^	1.23 ± 0.016^** h**^
OA (1 mM)	122.68 ± 0.80^**d**^	17.37 ± 0.02^**a**^	2.13 ± 0.016^**c**^	0.78 ± 0.011^**ab**^	4.46 ± 0.01^**ef**^	0.55 ± 0.003^**ef**^	3.92 ± 4.61^**b**^	0.48 ± 0.571^**d**^
OA (2 mM)	124.81 ± 0.20^**d**^	26.58 ± 0.01^**i**^	3.32 ± 0.007^**f**^	1.59 ± 0.022^**e**^	4.60 ± 0.01^** fg**^	0.57 ± 0.001^** h**^	10.49 ± 0.04^** k**^	1.31 ± 0.006^**i**^
OA (3 mM)	134.58 ± 2.00^**e**^	26.31 ± 0.05^**i**^	3.54 ± 0.046^** g**^	3.06 ± 0.162^**j**^	3.86 ± 0.01^**c**^	0.52 ± 0.007^**d**^	6.59 ± 0.01^**e**^	0.89 ± 0.012^**f**^

*AA* acetic acid, *CA* citric acid, *SA* succinic acid, *FA* fumaric acid, *OA* oxalic acid

Different superscript letters within columns indicate significant differences at p < 0.05

### Cyanobacterial growth modeling

The *A. platensis* growth under different culture conditions was fitted to the modified logistic model. The results confirmed a reasonable fitting of the modified logistic model to the experimental data as indicated by the high R^2^ and low RMSE values (Table 2).

**Table 2:** Kinetic modeling of *A. platensis* growth under different treatments using the modified logistic model

Treatment	µ_max_	Lag-time	RMSE	R^2^
Control	0.10 ± 0.012^a^	0.09 ± 0.005^a^	0.09	0.992
AA (1 mM)	0.15 ± 0.002^bc^	0.53 ± 0.024^bc^	0.07	0.976
AA (2 mM)	0.21 ± 0.025^gh^	1.90 ± 0.273^g^	0.10	0.989
AA (3 mM)	0.19 ± 0.005^efg^	1.51 ± 0.027^e^f	0.12	0.969
CA (1 mM)	0.15 ± 0.011^b^	0.01 ± 0.00^a^	0.09	0.961
CA (2 mM)	0.17 ± 0.007^bcd^	0.27 ± 0.104^ab^	0.13	0.954
CA (3 mM)	0.19 ± 0.010^defg^	0.05 ± 0.039^a^	0.11	0.969
SA (1 mM)	0.14 ± 0.000^b^	0.39 ± 0.238^abc^	0.09	0.971
SA (2 mM)	0.24 ± 0.006^i^	0.70 ± 0.271^c^	0.09	0.988
SA (3 mM)	0.20 ± 0.017^fgh^	0.29 ± 0.220^ab^	0.13	0.965
FA (1 mM)	0.18 ± 0.007^def^	1.10 ± 0.254^d^	0.10	0.980
FA (2 mM)	0.22 ± 0.000^hi^	1.43 ± 0.000^de^	0.08	0.989
FA (3 mM)	0.19 ± 0.001e^fg^	0.55 ± 0.148^bc^	0.12	0.975
OA (1 mM)	0.18 ± 0.013^cde^	1.16 ± 0.022^de^	0.08	0.982
OA (2 mM)	0.17 ± 0.004^bcd^	1.45 ± 0.045^de^	0.06	0.990
OA (3 mM)	0.18 ± 0.021^def^	1.85 ± 0.345^fg^	0.09	0.980

*AA* acetic acid, *CA* citric acid, *SA* succinic acid, *FA* fumaric acid, *OA* oxalic acid

Different superscript letters within columns indicate significant differences at p < 0.05

*µ*_*max*_ maximum growth rate, *RMSE* root means square error, *R*^*2*^ coefficient of determination

The fed-batch supplementation of different organic acids at different concentrations markedly increased the maximum growth rate (μ_max_) values in relation to the control, reflecting better growth and biomass production. The μ_max_ of control was 0.1 ± 0.012 day^−1^, which was increased to 0.14–0.24 day^−1^ in the organic acid-treated cultures (Table 2).

### Effect of different organic acids on phycobiliprotein’s content

Feeding the cyanobacterial culture with either succinic acid (3 mM), or oxalic acid (2 and 3 mM) every 3 days exhibited significantly higher PC contents (> 26 mg *g*^−1^) than other treatments (Table 1). This improvement was ~ 1.6-fold higher than the control (16.92 ± 0.44 mg *g*^−1^). However, it is worth mentioning here that most of the treatments promoted the PC contents of *A. platensis* to > 22 mg *g*^−1^, which was > 1.3-fold higher than the control. Similarly, the *A. platensis* cells exhibited higher APC contents in the presence of 2 mM of oxalic acid or 3 mM of succinic acid in the fed-batch culture. The enhancement of APC contents in these treatments was ~ 2.2-fold higher than the control (4.66 ± 0.26 mg *g*^−1^) (Table 1). On the other hand, the highest promotion of PE content was observed in the cultures incorporated with 2 mM citric acid, which was ~ 1.8-fold higher than the control (Table 1).

### Effect of different organic acids on phycobiliprotein’s productivity and PC purity

As listed in Table 1, the PC productivity was significantly enhanced by the supplementation of different organic acids compared to the control. The increase in PC productivity fluctuated between 1.3-fold and 3.08-fold higher than the control. The highest increase (26.70 ± 0.01 mg L^−1^ day^−1^) was observed in the cultures supplemented with 3 mM of succinic acid every 3-days treatment.

Most of the treatments showed better purity than control (Table 1). The highest values of the PC purity were observed in the culture treated with 3 mM of oxalic acid (purity 3.06 ± 0.16) or succinic acid (purity 2.20 ± 0.09) as well as 2 mM fumaric acid (purity 2.26 ± 0.10).

Similarly, succinic acid (3 mM) induced a marked increase in APC productivity to 1.70 ± 0.01 mg L^−1^ day^−1^, which was 4.36-fold higher than the control value (0.39 ± 0.01 mg L^−1^ day^−1^) (Table 1). Conversely, a 2.9-fold increase in PE productivity (0.84 mg L^−1^ day^−1^) was observed in 2 mM citric acid treatment in relation to the control (0.29 mg L^−1^ day^−1^) (Table 1).

### Effect of organic acids on the thermostability of phycocyanin

**Fig. 2:**
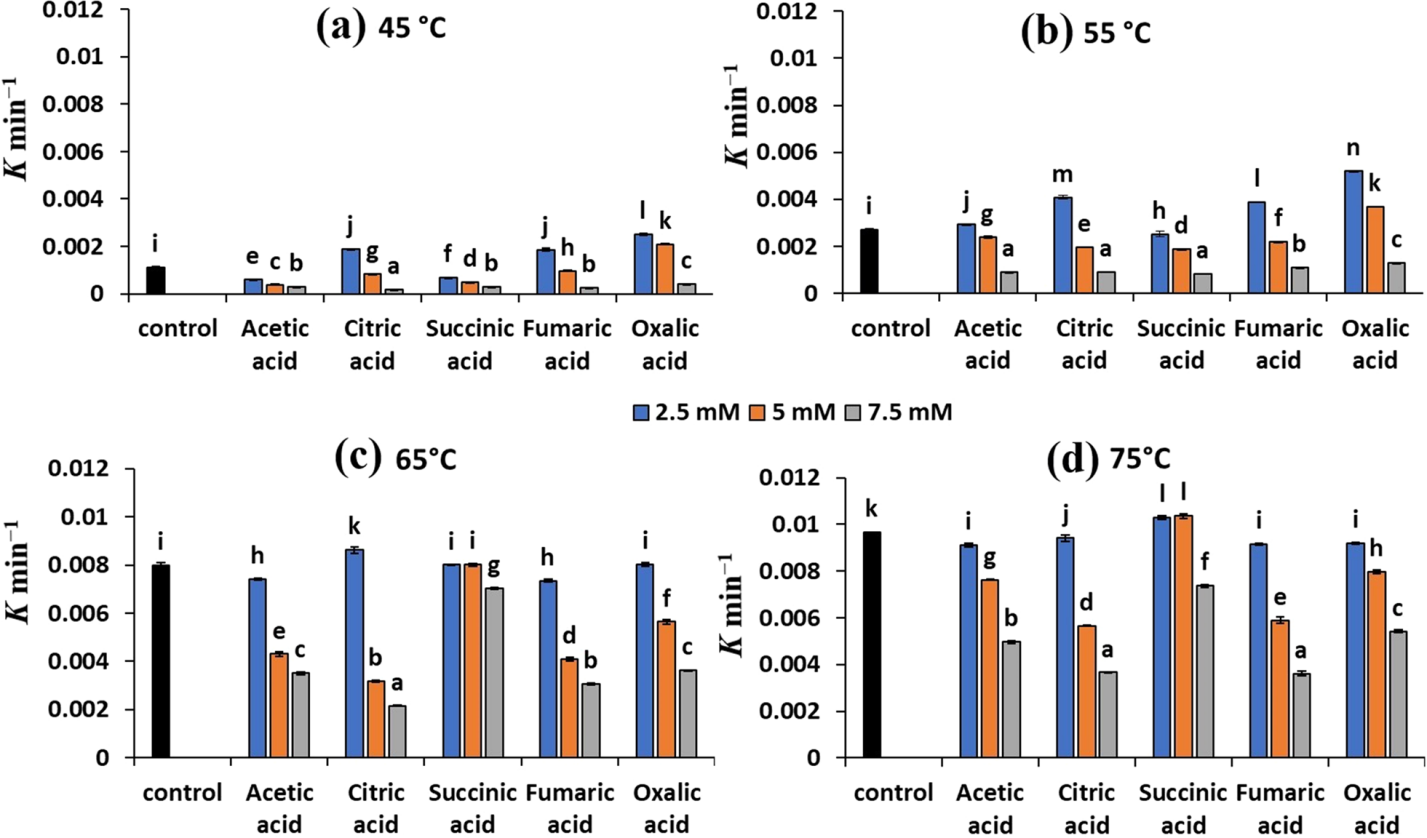
Variation in the degradation rate constant (*K*) values under different treatments of organic acids in relation to the control at different temperatures: **a** 45 ℃, **b** 55 ℃, **c** 65 ℃, and **d** 75 ℃. Different letters above columns indicate significant differences at p < 0.05

Different organic acids were investigated as chemical preservatives to promote the thermostability of the PC extract obtained from the fed-batch cultures of *A. platensis* supplemented with 3 mM succinic acid. The results indicated a remarkable decrease in the rate of degradation of PC by increasing the concentration of the investigated organic acids (Fig. 2).

At 45 ℃, the degradation rate of PC was reduced by ~ 84% with 7.5 mM of citric acid as a preservative (k = 0.00018 min^−1^) compared to the control (k = 0.00113 min^−1^) (Fig. 2a). While the use of citric acid, succinic acid, or acetic acid at a concentration of 7.5 mM for PC preservation at 55 ℃ showed to reduce the k values by ~ 70% compared the control (Fig. 2b). Similarly, citric acid at a concentration of 7.5 mM exhibited a significant reduction in the k values of PC by ~ 72% and 62% at 65 and 75 ℃ as compared to control, respectively (Fig. 2c, d). The use of fumaric acid in the stabilization of PC at 65 and 75 ℃ also exhibited a significant reduction in the k values of PC by ~ 62% compared to the control (Fig. 2d).

The half-life times (t_1/2_) also indicated a rapid degradation of PC in the control, while this process was slowed down by adding organic acids. The t_1/2_ of PC at 45 ℃ was 616.08 ± 15.52 min, which was markedly increased to 3827.83 ± 11.18 with 7.5 mM of citric acid (Table 3). While the use of succinic acid or acetic acid at 7.5 mM had increased the t_1/2_ of PC to 2383.62 ± 11.59 and 2372.18 ± 8.04 min at 45 ℃, respectively (Table 3). Conversely, at 55 ℃, the highest increase in the t_1/2_ of PC was obtained with 7.5 mM of succinic acid (836.58 ± 3.21 min), followed by 7.5 mM of acetic acid (771.76 ± 3.83 min) and 7.5 mM of citric acid (767.85 ± 4.13 min) in relation to the control (257.23 ± 6.55 min) (Table 3). Conversely, citric acid (7.5 mM) and fumaric acid (7.5 mM) exhibited significant promotion of the t_1/2_ of PC in relation to other concentrations of organic acids at both 65 and 75 ℃. The t_1/2_ of PC at 65 ℃ was increased to 321.06 ± 2.22 min with citric acid (7.5 mM) and to 226.68 ± 2.66 min with fumaric acid (7.5 mM) compared to the control (86.73 ± 0.95 min) (Table 3). Similarly, at 75 ℃, the t_1/2_ of PC was enhanced to 189.44 ± 1.07 and 191.93 ± 5.80 with citric acid (7.5 mM) and fumaric acid (7.5 mM), respectively compared to 71.84 ± 0.20 without preservatives (Table 3).

**Table 3:** Half-life times (t1/2) of phycocyanin under different temperatures in the presence of different concentrations of organic acids as preservatives

Treatment	45 ℃		55 ℃		65 ℃		75 ℃	
	t_1/2_	R^2^	t_1/2_	R^2^	t_1/2_	R^2^	t_1/2_	R^2^
Control	616.08 ± 15.52^d^	0.90	257.23 ± 6.55^e^	0.97	86.73 ± 0.95^b^	0.97	71.84 ± 0.20^b^	0.90
AA (2.5 mM)	1151.89 ± 3.38^ h^	0.98	236.53 ± 0.05^d^	0.93	93.54 ± 0.70^c^	0.92	76.07 ± 0.54^c^	0.90
AA (5 mM)	1770.28 ± 7.99^j^	0.95	290.55 ± 8.07^ g^	0.94	161.29 ± 3.00^f^	0.9	90.95 ± 0.32^e^	0.97
AA (7.5 mM)	2372.18 ± 8.04^ k^	0.95	771.76 ± 3.83^ m^	0.9	197.79 ± 3.29^i^	0.91	139.76 ± 1.69^i^	0.89
CA (2.5 mM)	369.03 ± 0.39^c^	0.99	169.64 ± 3.29^b^	0.99	80.42 ± 1.26^a^	0.99	73.61 ± 1.04b^c^	0.94
CA (5 mM)	834.36 ± 8.81^f^	0.88	351.79 ± 3.93^i^	0.95	218.70 ± 2.50^j^	0.97	122.62 ± 0.49^ g^	0.97
CA (7.5 mM)	3827.83 ± 11.18^ m^	0.96	767.85 ± 4.13^ m^	0.97	321.06 ± 2.22^ l^	0.99	189.44 ± 1.07^j^	0.91
SA (2.5 mM)	1020.67 ± 11.36^ g^	0.99	274.99 ± 10.85^f^	0.99	86.60 ± 0.10^b^	0.91	67.38 ± 0.59^a^	0.90
SA (5 mM)	1380.18 ± 10.13^i^	0.96	371.66 ± 4.78^j^	0.95	86.53 ± 0.70^b^	0.88	66.90 ± 0.67^a^	0.88
SA (7.5 mM)	2383.62 ± 11.59^ k^	0.95	836.58 ± 3.21^n^	0.99	98.70 ± 0.59^d^	0.99	94.10 ± 0.70^e^	0.96
FA (2.5 mM)	372.23 ± 9.98^c^	0.95	178.12 ± 0.31^i^	0.96	94.30 ± 0.86^c^	0.96	75.67 ± 0.30^bc^	0.96
FA (5 mM)	717.87 ± 9.41^e^	0.98	317.53 ± 4.78^ h^	0.98	169.41 ± 3.25^ g^	0.97	117.54 ± 3.00^f^	0.98
FA (7.5 mM)	2723.03 ± 0.76^ l^	0.99	639.85 ± 6.22^bc^	0.94	226.68 ± 2.66^ k^	0.97	191.93 ± 5.80^j^	0.90
OA (2.5 mM)	276.54 ± 2.97^a^	0.99	133.58 ± 0.81^a^	0.98	86.39 ± 0.63^b^	0.99	75.55 ± 0.28^bc^	0.95
OA (5 mM)	330.68 ± 3.97^b^	0.97	188.36 ± 1.66^c^	0.99	123.02 ± 2.14^e^	0.99	87.04 ± 2.14^d^	0.97
OA (7.5 mM)	1754.37 ± 6.28^j^	0.94	539.72 ± 3.57^ k^	0.97	190.46 ± 0.24^ h^	0.97	127.88 ± 1.36^ h^	0.97

R^2^: coefficient of determination of the plot ln *(c*_*t*_*/c*_*0*_*)* vs.* t*

*AA* acetic acid, *CA* citric acid, *SA* succinic acid, *FA* fumaric acid, *OA* oxalic acid

Different superscript letters within columns indicate significant differences at p < 0.05

### Thermodynamics of PC inactivation

The mechanism of PC denaturation at different temperatures in the solution with different organic acids and without preservatives was evaluated using different thermodynamic parameters. The ∆H° values obtained for PC at different treatments and temperatures were always positive and slightly increased with increasing the concentration of the preservative from 2.5 to 7.5 mM (Table 4). The ∆H° of PC in the absence of preservatives was 66.88 kJ mol^−1^. A marked increase in ∆H° of PC was observed in the presence of different organic acids at 7.5 mM, but the highest values were related to succinic acid (∆H° = 106.59 kJ mol^−1^) followed by citric acid (∆H° = 88.78 kJ mol^−1^) and acetic acid (∆H° = 88.30 kJ mol^−1^) (Table 4).

**Table 4:** Evaluated thermodynamic parameters during the inactivation of phycocyanin in the presence of different preservatives in relation to the control

Treatment	E (kJ mol^−1^)	∆H° (kJ mol^−1^)	∆S° (kJ mol^−1^)	∆G (kJ mol^−1^)
Control	69.65	66.88	− 0.087	94.42 − 97.02
AA (2.5 mM)	84.25	81.49	− 0.044	95.51 − 96.83
AA (5 mM)	88.00	85.23	− 0.036	96.57 − 97.64
AA (7.5 mM)	91.06	88.30	− 0.030	97.96 − 98.88
CA (2.5 mM)	51.71	48.95	− 0.138	92.99 − 97.14
CA (5 mM)	57.43	54.67	− 0.128	95.26 − 99.09
CA (7.5 mM)	91.55	88.78	− 0.032	98.82 − 99.77
SA (2.5 mM)	86.17	83.42	− 0.038	95.48 − 96.62
SA (5 mM)	97.02	94.25	− 0.007	96.80 − 96.99
SA (7.5 mM)	107.90	105.14	0.022	98.10 − 97.44
FA (2.5 mM)	50.08	47.31	− 0.144	93.07 − 97.39
FA (5 mM)	55.95	53.19	− 0.131	94.83 − 98.76
FA (7.5 mM)	83.42	80.66	− 0.054	94.83 − 99.52
OA (2.5 mM)	40.13	37.37	− 0.172	92.19 − 97.36
OA (5 mM)	40.86	38.10	− 0.172	92.90 − 98.07
OA (7.5 mM)	82.25	79.49	− 0.055	97.06 − 98.72

*AA* acetic acid, *CA* citric acid, *SA* succinic acid, *FA* fumaric acid, *OA* oxalic acid

Different superscript letters within columns indicate significant differences at p < 0.05

The ∆S° values of PC were negative and was calculated to be –0.087 kJ mol^−1^ before the addition of any organic acid (Table 4). The use of citric acid, fumaric acid, and organic acid at 2.5 or 5 mM showed to decrease the ∆S° values of PC to < –0.128 kJ mol^−1^ (Table 4). Conversely, increasing the concentration of these organic acids to 7.5 mM exhibited a marked increase of the ∆S° values of PC to > –0.055 kJ mol^−1^. On the other hand, the application of acetic acid and succinic acid was prominent in increasing the ∆S° values of PC at different tested concentrations in relation to the control, and the highest values were obtained for succinic acid at 5 and 7.5 mM (Table 4).

On the other hand, the ∆G° values of PC inactivation were always positive at different treatments (Table 4). The incorporation of organic acids elevated the ∆G° values of PC, especially at 7.5 mM. The highest increase in the ∆G° values of PC in relation to the control was observed with 7.5 mM of citric acid (98.82–99.77 kJ mol^−1^) (Table 4).

The activation energy (E) of PC before adding organic acids was 69.65 kJ mol^−1^) (Table 4). Succinic acid and citric acid at different investigated concentrations markedly increased the E values of PC. The E values were increased to 91.06 and 107.90 kJ mol^−1^ in the presence of 7.5 mM of acetic acid and succinic acid, respectively (Table 4).

## Discussion

Different organic acids such as citric acid, succinic acid, fumaric acid, and oxalic acid are well known intermediates of the tricarboxylic acid (TCA) cycle. However, these acids have hormesis effects on microalgae; the low concentrations can promote microalgal growth and metabolism, while higher concentrations exhibit strong algicidal activity [8, 9]. Accordingly, microalgae can utilize organic acids as a source of carbon, and their incorporation into the culture medium under fed-batch mode can minimize the toxic effects of these compounds. The results of the present study confirmed that the use of organic acids as chemical stimulants at proper concentrations during fed-batch cultivation of *A. platensis* could be a promising solution to get the better of biomass production and promote the accumulation of phycobiliproteins. Feeding the cyanobacterial culture every 3-days with 2–3 mM of either fumaric acid, succinic acid and acetic acid almost doubled the biomass productivity after 12 days compared to the control. The incorporation of succinic acid at 1–10 mM into the Zarrouk’s medium have been reported to promote biomass production of *A. platensis* [9]*.* Similarly, an enhancement of the biomass production of the green microalga *Haematococcus pluvialis* was observed using fed-batch cultivation using succinic acid [10]. This promotion in cell growth may be attributed mainly to the simultaneous utilization of organic acids as a source of organic carbon and CO_2_ as an inorganic carbon for cellular growth and metabolism. This mixotrophic nutrition has been reported to give superior biomass yield compared to photoautotrophic nutrition [23]. The incorporation of succinic acid into microalgal cultures was associated with the upregulation of ribulose-1,5-bisphosphate carboxylase/oxygenase (Rubisco), which can increase the photosynthetic rates with a subsequent increase in cell growth [24].

Similarly, the organic acid fed-batch growth of *A. platensis* induced the accumulation of phycobiliproteins. The highest PC productivity in the present study reached 4.38 mg L^−1 ^day^−1^ in the 3 mM of succinic acid treatment. This treatment also exhibited higher APC productivity, but the highest PE productivity was observed at 2 mM of citric acid. The enhancement of PC content and productivity in the current study was consistent with the observations of [9]. This enhancement was attributed to the overexpression of the genes associated with PC biosynthesis in *A. platensis* grown in the presence of succinic acid*.* In another study, fed-batch cultivation of *H. pluvialis* using succinic acid boosted its pigment (astaxanthin) content [10]. This study also reported an increase in the reactive oxygen species in the microalgal culture supplemented with organic acids. Accordingly, the increase in pigment contents is one of the main mechanisms to alleviate the oxidative stress on the microalgal cells [25]. The algicidal activity of different organic acids on the toxic cyanobacterium *Microcystis* sp. have been associated with 27-fold increase in PC contents [8].

According to purity ratio, PC extract is classified into food grade (purity ratio around 0.7), reactive grade (purity ratio around 3.9), and analytical grade (purity ratio > 4.0) [26]. The purity ratio of PC in the present study is comparatively as high as the food grade value, which implied its potential application in the preparation of high-quality food products. Furthermore, the fed-batch cultivation using organic acids showed to improve the purity ratio of the PC extract compared to the control.

Denaturation of protein moieties in PC has marked effects on its color stability [27]. PC is highly prone to degradation with a loss of ~ 90–95% upon exposure to environmental factors such as light, temperature, pH and O_2_ [20]. Thus, chemical substances such as glucose, sucrose, citric acid, sorbic acid, NaCl, ascorbic acid, and NaN_3_ have been reported as common preservatives to stabilize the color of PC and extend its shelf-life [27]. The present results were consistent with previous studies on PC from *A. platensis* which indicated a degradation of more than 60% at higher temperatures [26]. The use of different organic acids as edible preservatives at proper concentration could improve the thermostability of PC. The crude PC extract showed better thermal stability in the presence of citric acid at 7.5 mM in relation to other treatments. This result agreed with previous studies which identified citric acid as one of the most effective preservatives for PC [14]. The t_1/2_ values of PC in the presence of 7.5 mM citric acid at 45, 55, 65, and 75 ℃ were 3827.83, 767.85, 321.06, and 189.44 min, respectively. Furthermore, after 90 min at these temperatures, citric acid preserved more than 70% of PC concentration compared to about 30% without preservatives. Similarly, citric acid (0.1 *g*) have been reported to preserve about 70% of PC after 45 days at 35 ℃ [11]. In another study, about 19% of PC was remained after 60 min treatment at 80 ℃ in the presence of citric acid (0.4%) [14]. Generally, citric acid is a natural, renewable, and non-toxic substance, which is commonly used in the food industry. It acts as a chelator and possesses strong antioxidant activity. The importance of organic acids in stabilizing PC at high temperatures may be related to the formation of covalent and/or non-covalent interactions between the protein and the acid [28].

The variation in enthalpy (△H°) during thermal inactivation reflects the amount of energy required for denaturing protein moiety of PC. Thus, higher △H° values are indicative of higher protein thermostability. All the investigated organic acids were prominent in increasing △H° of PC especially at higher concentrations with succinic acid (7.5 mM) being the most efficient (△H° = 106.59 kJ mol^−1^) followed by 7.5 mM citric acid (△H° = 88.78 kJ mol^−1^) in relation to the control (△H° = 66.88 kJ mol^−1^). Similarly, Wan and coworkers observed an increase of △H° value during thermal denaturation of PC from *A. platensis* by increasing sodium citrate as a preservative from 0.5 to 5 mM [29]. Moreover, the positive △H° values manifested that the thermal denaturation of PC is endothermic [30]. On the other hand, the thermal denaturation of PC results in higher disordering of the protein structure, thus the entropy (△S°) increases. The △S° values of PC inactivation in most of the treatments were negative, which reflected that the native protein moiety is more in the ordered state [31]. Furthermore, it manifested the occurrence of protein aggregation rather than unfolding during the thermal denaturation process [30]. In general, the positive △H° and negative △S° reflected that the inactivation process is not spontaneous at any temperature [30]. On the other side, Gibb’s free energy (△G°) is a more reliable measurement of thermal stability, with higher values are indicative of higher stability. The △G° values for PC extract were increased in most of the treatments compared to the control. Additionally, the △G° values exhibited a small increase with increasing the temperature, reflecting a degree of thermostability. The highest values for △G° were observed in the 7.5 mM citric acid treatment, which reflected higher thermal stabilization of PC in this treatment. This result supports the observations of the kinetic study.

## Conclusion

The present study highlighted the effectiveness of different organic acids in the biomass and phycobiliproteins production from *A. platensis.* Succinic acid (3 mM) was identified as the best organic acid to markedly promote cyanobacterial biomass and PC productivity under fed-batch cultivation. The PC productivity in this treatment reached about threefold higher than the control, and its purity ratio was 2.20, suggesting a promising food-grade product. On the other hand, the investigated organic acids were evaluated as potential preservatives to stabilize PC at higher temperatures. Citric acid (7.5 mM) was identified as the best preservative in relation to other organic acids to promote thermostability of PC at higher temperatures. The t_1/2_ of PC at 75 ℃ was promoted to 189.44 min compared to 71.84 min in the control. The thermodynamic analysis further confirmed the thermostability of PC in the presence of organic acids and indicated the endothermic and non-spontaneity of the thermal denaturation process. The findings of the present study confirmed the dual functional properties of organic acids as cost effective and sustainable compounds for promoting not only phycobiliproteins’ production but also the thermostability of PC for potential application in food industry.

## Data Availability

The datasets used and/or analyzed during the current study are available from the corresponding author on reasonable request.
